# Supplementary figures and images for: Comparative Transcriptome Analysis Reveals Key Insights into Fertility Conversion in the Thermo-Sensitive Cytoplasmic Male Sterile Wheat
Source: Int J Mol Sci. 2022 Nov 18;23(22):14354. doi: 10.3390/ijms232214354 (PMC9693999; doi:10.3390/ijms232214354)

Supplementary figure

Figure S1. KOG functional annotation classification of DEGs.

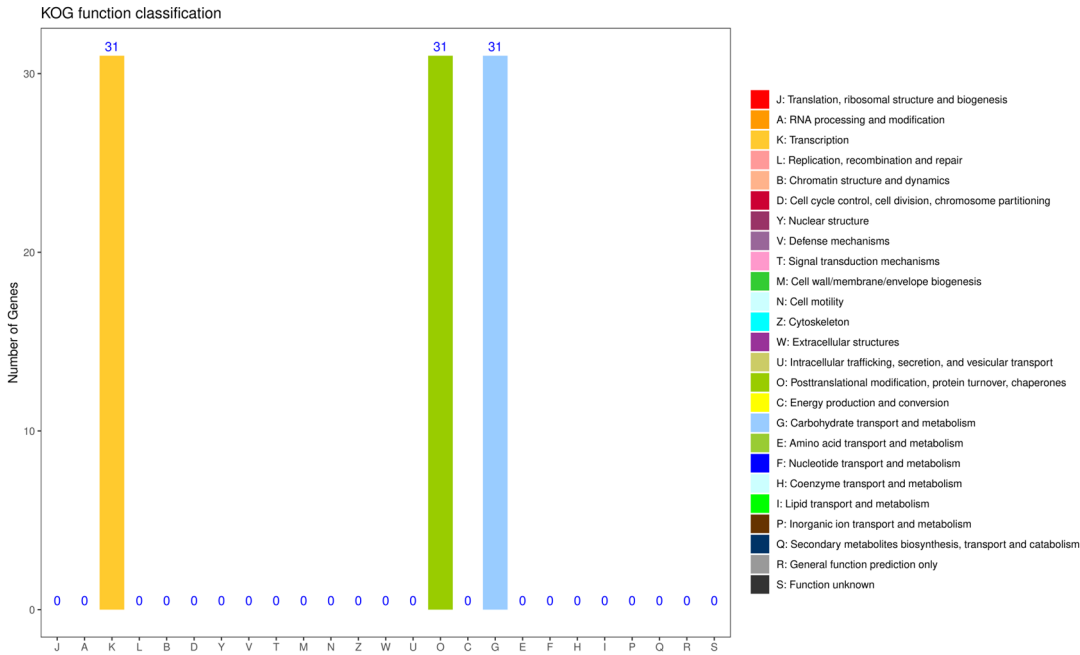

Supplement: Supplementary file 1 [file ijms-23-14354-s001.zip › Supplementary figure.pdf]
